# Neptunium(III) copper(I) diselenide

**DOI:** 10.1107/S160053680900395X

**Published:** 2009-02-11

**Authors:** Daniel M. Wells, S. Skanthakumar, L. Soderholm, James A. Ibers

**Affiliations:** a Chemistry Department, Northwestern University, 2145 Sheridan Rd, Evanston, IL 60208, USA; b Chemistry Division, Argonne National Laboratory, Argonne, IL 60439, USA

## Abstract

The title compound, NpCuSe_2_, is the first ternary neptunium transition-metal chalcogenide. It was synthesized from the elements at 873 K in an evacuated fused-silica tube. Single crystals were grown by vapor transport with I_2_. NpCuSe_2_ crystallizes in the LaCuS_2_ structure type and can be viewed as a stacking of layers of CuSe_4_ tetra­hedra and of double layers of NpSe_7_ monocapped trigonal prisms along [100]. Because there are no Se—Se bonds in the structure, the formal oxidation states of Np/Cu/Se may be assigned as +III/+I/−II, respectively.

## Related literature

For discussion of the LaCuS_2_ structure type, see: Julien-Pouzol *et al.* (1981 ▶); Ijjaali *et al.* (2004 ▶). For other compounds with Cu—Se bonds, see: Daoudi *et al.* (1996 ▶); Strobel & Schleid (2004 ▶); Ijjaali *et al.* (2004 ▶). For other neptunium selenides, see: Wastin *et al.* (1995 ▶); Wojakowski (1985 ▶). For computational details, see Gelato & Parthé (1987 ▶).

## Experimental

### 

#### Crystal data

NpCuSe_2_
*M* *_r_* = 458.46
Monoclinic, 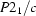

*a* = 6.6796 (5) Å
*b* = 7.4384 (6) Å
*c* = 7.1066 (5) Å
β = 97.156 (1)°
*V* = 350.34 (5) Å^3^
*Z* = 4
Mo *K*α radiation
μ = 56.06 mm^−1^
*T* = 100 (2) K
0.08 × 0.05 × 0.04 mm

#### Data collection

Bruker APEXII CCD diffractometer
Absorption correction: numerical (face indexed; *SADABS*; Sheldrick, 2006 ▶) *T*_min_ = 0.045, *T*_max_ = 0.212
6189 measured reflections
1376 independent reflections
1309 reflections with *I* > 2σ(*I*)
*R*_int_ = 0.036

#### Refinement

*R*[*F* ^2^ > 2σ(*F* ^2^)] = 0.028
*wR*(*F* ^2^) = 0.068
*S* = 1.35
1376 reflections
37 parameters
Δρ_max_ = 2.43 e Å^−3^
Δρ_min_ = −4.48 e Å^−3^

### 

Data collection: *APEX2* (Bruker, 2006 ▶); cell refinement: *SAINT* (Bruker, 2006 ▶); data reduction: *SAINT*; program(s) used to solve structure: *SHELXS97* (Sheldrick, 2008 ▶); program(s) used to refine structure: *SHELXL97* (Sheldrick, 2008 ▶); molecular graphics: *CrystalMaker* (Palmer, 2008 ▶); software used to prepare material for publication: *SHELXTL* (Sheldrick, 2008 ▶).

## Supplementary Material

Crystal structure: contains datablocks I, global. DOI: 10.1107/S160053680900395X/wm2219sup1.cif

Structure factors: contains datablocks I. DOI: 10.1107/S160053680900395X/wm2219Isup2.hkl

Additional supplementary materials:  crystallographic information; 3D view; checkCIF report

## Figures and Tables

**Table:** 

Cu1—Se2^i^	2.4409 (9)
Cu1—Se1^ii^	2.4490 (9)
Cu1—Se1^iii^	2.5066 (9)
Cu1—Se1	2.5899 (9)
Np1—Se2^iv^	2.9330 (6)
Np1—Se2^v^	2.9540 (6)
Np1—Se2	2.9743 (6)
Np1—Se1^vi^	2.9784 (6)
Np1—Se2^vii^	2.9785 (6)
Np1—Se1	2.9950 (6)
Np1—Se1^viii^	3.1419 (6)

**Table:** 

Se2^i^—Cu1—Se1^ii^	116.52 (4)
Se2^i^—Cu1—Se1^iii^	102.85 (3)
Se1^ii^—Cu1—Se1^iii^	112.76 (4)
Se2^i^—Cu1—Se1	103.94 (3)
Se1^ii^—Cu1—Se1	103.44 (3)

Symmetry codes: (i) 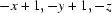; (ii) 
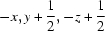
; (iii) 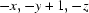; (iv) 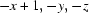; (v) 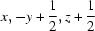; (vi) 
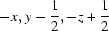
; (vii) 
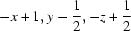
; (viii) 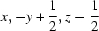.

**Fig. 1.**
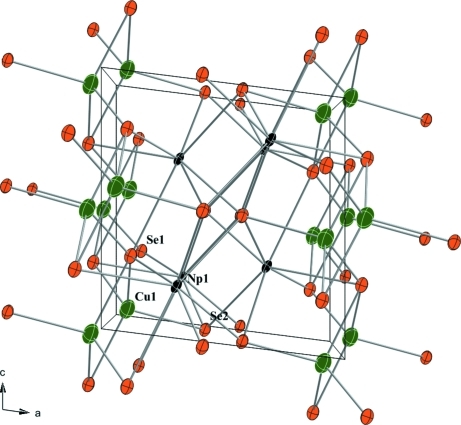
A view nearly down [010] of the unit cell of NpCuSe2, with displacement ellipsoids at the 99% probability level.

## supplementary crystallographic information

### Comment

In keeping with earlier descriptions of the LaCuS_2_ structure type (Julien-Pouzol *et al.*, 1981; Ijjaali *et al.*, 2004) the structure of NpCuSe_2_ can be viewed as a stacking of layers of CuSe_4_ tetrahedra and double layers of NpSe_7_ monocapped trigonal prisms along [100]. Figure 1 provides a view nearly down [010] of the unit cell. It displays the stacking of layers along [100] where atom Se1 is contained within the Cu layer and atom Se2 is contained within the Np double layer. The Cu—Se bond distances are reasonable for a Cu(I) compound; they range from 2.4409 (9) to 2.5899 (9) Å compared to 2.458 (2) to 2.490 (4) Å in SrCuCeSe_3_ (Strobel & Schleid, 2004) and 2.450 (1) to 2.607 (1) Å in the Ce analogue CeCuSe_2_ (Ijjaali *et al.*, 2004). The Np—Se bond distances range from 2.9330 (6) to 3.1419 (6) Å. Comparisons are limited but can be made with the Np—Se distance of 2.903 (1) Å in NpSe (Wastin *et al.*, 1995) and those of 2.932 and 3.086 Å in NpAsSe (Wojakowski, 1985). There are no Se—Se bonds in NpCuSe_2_, so formal oxidation states may be assigned for Np/Cu/Se of +III/+I/-II.

The chemistry of Np is transitional between that of U and Pu. All three elements exhibit multiple oxidation states in their compounds. NpCuSe_2_ is the first example of a neptunium chalcogenide compound analogous to a lanthanide(III) structure rather than to a transition-metal or uranium(IV) structure. The Pu analogue is unknown, although arguments based on the stability of various Pu oxidation states suggest it should be stable.

### Experimental

NpCuSe_2_was formed in an attempted synthesis of the Np analogue of U_3_Cu_2_Se_7_ (Daoudi *et al.*, 1996). Caution! ^237^Np is an α-emitting radioisotope and as such is considered a health risk. Its use requires appropriate infrastructure and personnel trained in the handling of radioactive materials. The following reagents were used as obtained from the manufacturer: Cu (Aldrich, 99.5%) and Se (Aldrich, 99%). Resublimed I_2_ was utilized as a transport reagent. ^237^Np chunks were crushed and used as provided from Oak Ridge National Laboratory. A reaction mixture of 20.2 mg Np (0.085 mmol), 3.58 mg Cu (0.056 mmol), and 15.55 mg Se (0.197 mmol) was loaded into a fused-silica ampoule in an Ar-filled dry box that was then evacuated to 10 ^-4^ Torr and sealed. The sample was placed in a computer controlled furnace, heated to 873 K in 8 h, kept at 873 K for 72 h, cooled at 5 K/h to 373 K, and finally air cooled in the oven to 298 K. The resultant black powder was reloaded into a fused-silica ampoule with 4 mg I_2_. The ampoule was evacuated to 10 ^-4^ Torr and sealed. The sample was placed in a computer controlled furnace, heated to 873 K in 8 h, kept at 873 K for 336 h, cooled at 6.94 K/h to 373 K, before finally being air cooled to 298 K. Black rectangular plates and blocks of NpCuSe_2_ were obtained in low yield. The crystals used in characterization were manually extracted from the product mixture.

### Refinement

The program *STRUCTURE TIDY* (Gelato & Parthé, 1987) was employed to standardize the atomic coordinates of the structure. The highest peak is 1.71 Å and the deepest hole is 0.08 Å from atom Np1.

### Crystal data

**Table:**

NpCuSe_2_	*F*(000) = 760
*M**_r_* = 458.46	*D*_x_ = 8.692 Mg m^−^^3^
Monoclinic, *P*2_1_/*c*	Mo *K*α radiation, λ = 0.71073 Å
Hall symbol: -P 2ybc	Cell parameters from 4110 reflections
*a* = 6.6796 (5) Å	θ = 4.0–33.7°
*b* = 7.4384 (6) Å	µ = 56.06 mm^−^^1^
*c* = 7.1066 (5) Å	*T* = 100 K
β = 97.156 (1)°	Block, black
*V* = 350.34 (5) Å^3^	0.08 × 0.05 × 0.04 mm
*Z* = 4	

### Data collection

**Table:**

Bruker APEXII CCD diffractometer	1376 independent reflections
Radiation source: fine-focus sealed tube	1309 reflections with *I* > 2σ(*I*)
graphite	*R*_int_ = 0.036
φ and ω scans	θ_max_ = 33.9°, θ_min_ = 3.1°
Absorption correction: numerical (face indexed; *SADABS*; Sheldrick, 2006)	*h* = −10→10
*T*_min_ = 0.045, *T*_max_ = 0.212	*k* = −11→11
6189 measured reflections	*l* = −11→11

### Refinement

**Table:**

Refinement on *F*^2^	0 restraints
Least-squares matrix: full	Primary atom site location: structure-invariant direct methods
*R*[*F*^2^ > 2σ(*F*^2^)] = 0.028	Secondary atom site location: difference Fourier map
*wR*(*F*^2^) = 0.068	*w* = [1/[σ^2^(*F*_o_^2^) + (0.0312)*F*_o_^2^]^2^
*S* = 1.35	(Δ/σ)_max_ < 0.001
1376 reflections	Δρ_max_ = 2.43 e Å^−^^3^
37 parameters	Δρ_min_ = −4.48 e Å^−^^3^

### Fractional atomic coordinates and isotropic or equivalent isotropic displacement parameters (Å^2^)

**Table:**

	*x*	*y*	*z*	*U*_iso_*/*U*_eq_	
Cu1	0.07000 (11)	0.66155 (10)	0.04945 (11)	0.00850 (14)	
Np1	0.30684 (3)	0.04823 (3)	0.19759 (3)	0.00478 (8)	
Se1	0.09977 (8)	0.39107 (7)	0.28075 (8)	0.00539 (11)	
Se2	0.58173 (9)	0.27585 (7)	0.00026 (8)	0.00520 (11)	

### Atomic displacement parameters (Å^2^)

**Table:**

	*U*^11^	*U*^22^	*U*^33^	*U*^12^	*U*^13^	*U*^23^
Cu1	0.0082 (3)	0.0081 (3)	0.0091 (3)	−0.0008 (2)	0.0009 (2)	−0.0017 (2)
Np1	0.00560 (11)	0.00354 (11)	0.00509 (11)	−0.00032 (6)	0.00021 (8)	−0.00020 (6)
Se1	0.0063 (2)	0.0041 (2)	0.0056 (2)	0.00028 (16)	−0.00019 (18)	−0.00004 (17)
Se2	0.0058 (2)	0.0044 (2)	0.0051 (2)	0.00028 (17)	−0.00027 (18)	0.00013 (17)

### Geometric parameters (Å, °)

**Table:**

Cu1—Se2^i^	2.4409 (9)	Np1—Se1	2.9950 (6)
Cu1—Se1^ii^	2.4490 (9)	Np1—Se1^v^	3.1419 (6)
Cu1—Se1^iii^	2.5066 (9)	Np1—Cu1^viii^	3.3772 (8)
Cu1—Se1	2.5899 (9)	Np1—Cu1^x^	3.3866 (8)
Cu1—Cu1^iii^	2.6421 (15)	Np1—Cu1^vii^	3.4894 (8)
Cu1—Np1^ii^	3.3772 (8)	Se1—Cu1^viii^	2.4490 (9)
Cu1—Np1^iv^	3.3866 (8)	Se1—Cu1^iii^	2.5066 (9)
Cu1—Np1^v^	3.4894 (8)	Se1—Np1^ii^	2.9784 (6)
Np1—Se2^vi^	2.9330 (6)	Se1—Np1^vii^	3.1419 (6)
Np1—Se2^vii^	2.9540 (6)	Se2—Cu1^i^	2.4409 (9)
Np1—Se2	2.9743 (6)	Se2—Np1^vi^	2.9330 (6)
Np1—Se1^viii^	2.9784 (6)	Se2—Np1^v^	2.9540 (6)
Np1—Se2^ix^	2.9785 (6)	Se2—Np1^xi^	2.9785 (6)
			
Se2^i^—Cu1—Se1^ii^	116.52 (4)	Se2^vi^—Np1—Cu1^viii^	135.297 (18)
Se2^i^—Cu1—Se1^iii^	102.85 (3)	Se2^vii^—Np1—Cu1^viii^	86.489 (18)
Se1^ii^—Cu1—Se1^iii^	112.76 (4)	Se2—Np1—Cu1^viii^	130.824 (18)
Se2^i^—Cu1—Se1	103.94 (3)	Se1^viii^—Np1—Cu1^viii^	47.587 (17)
Se1^ii^—Cu1—Se1	103.44 (3)	Se2^ix^—Np1—Cu1^viii^	85.522 (17)
Se1^iii^—Cu1—Se1	117.57 (3)	Se1—Np1—Cu1^viii^	44.705 (16)
Se2^i^—Cu1—Cu1^iii^	116.56 (4)	Se1^v^—Np1—Cu1^viii^	101.316 (18)
Se1^ii^—Cu1—Cu1^iii^	126.51 (5)	Se2^vi^—Np1—Cu1^x^	44.728 (17)
Se1^iii^—Cu1—Cu1^iii^	60.33 (3)	Se2^vii^—Np1—Cu1^x^	145.739 (18)
Se1—Cu1—Cu1^iii^	57.24 (3)	Se2—Np1—Cu1^x^	128.963 (18)
Se2^i^—Cu1—Np1^ii^	155.73 (3)	Se1^viii^—Np1—Cu1^x^	44.687 (17)
Se1^ii^—Cu1—Np1^ii^	59.35 (2)	Se2^ix^—Np1—Cu1^x^	73.231 (18)
Se1^iii^—Cu1—Np1^ii^	100.32 (3)	Se1—Np1—Cu1^x^	125.113 (18)
Se1—Cu1—Np1^ii^	58.107 (19)	Se1^v^—Np1—Cu1^x^	72.255 (17)
Cu1^iii^—Cu1—Np1^ii^	69.64 (3)	Cu1^viii^—Np1—Cu1^x^	91.556 (15)
Se2^i^—Cu1—Np1^iv^	57.74 (2)	Se2^vi^—Np1—Cu1^vii^	98.092 (18)
Se1^ii^—Cu1—Np1^iv^	58.79 (2)	Se2^vii^—Np1—Cu1^vii^	88.423 (18)
Se1^iii^—Cu1—Np1^iv^	124.26 (3)	Se2—Np1—Cu1^vii^	162.568 (18)
Se1—Cu1—Np1^iv^	117.81 (3)	Se1^viii^—Np1—Cu1^vii^	44.741 (17)
Cu1^iii^—Cu1—Np1^iv^	172.48 (5)	Se2^ix^—Np1—Cu1^vii^	43.454 (17)
Np1^ii^—Cu1—Np1^iv^	113.38 (2)	Se1—Np1—Cu1^vii^	88.717 (17)
Se2^i^—Cu1—Np1^v^	57.06 (2)	Se1^v^—Np1—Cu1^vii^	123.660 (17)
Se1^ii^—Cu1—Np1^v^	160.72 (3)	Cu1^viii^—Np1—Cu1^vii^	45.22 (2)
Se1^iii^—Cu1—Np1^v^	56.76 (2)	Cu1^x^—Np1—Cu1^vii^	66.861 (13)
Se1—Cu1—Np1^v^	95.83 (3)	Cu1^viii^—Se1—Cu1^iii^	99.74 (3)
Cu1^iii^—Cu1—Np1^v^	65.14 (3)	Cu1^viii^—Se1—Cu1	148.28 (3)
Np1^ii^—Cu1—Np1^v^	134.78 (2)	Cu1^iii^—Se1—Cu1	62.43 (3)
Np1^iv^—Cu1—Np1^v^	111.51 (2)	Cu1^viii^—Se1—Np1^ii^	76.53 (2)
Se2^vi^—Np1—Se2^vii^	122.772 (13)	Cu1^iii^—Se1—Np1^ii^	78.50 (2)
Se2^vi^—Np1—Se2	91.915 (16)	Cu1—Se1—Np1^ii^	74.31 (2)
Se2^vii^—Np1—Se2	74.152 (12)	Cu1^viii^—Se1—Np1	75.95 (2)
Se2^vi^—Np1—Se1^viii^	89.408 (17)	Cu1^iii^—Se1—Np1	81.29 (2)
Se2^vii^—Np1—Se1^viii^	128.634 (17)	Cu1—Se1—Np1	122.48 (3)
Se2—Np1—Se1^viii^	150.258 (17)	Np1^ii^—Se1—Np1	142.27 (2)
Se2^vi^—Np1—Se2^ix^	74.394 (9)	Cu1^viii^—Se1—Np1^vii^	79.17 (2)
Se2^vii^—Np1—Se2^ix^	72.516 (18)	Cu1^iii^—Se1—Np1^vii^	178.90 (3)
Se2—Np1—Se2^ix^	127.853 (11)	Cu1—Se1—Np1^vii^	118.47 (3)
Se1^viii^—Np1—Se2^ix^	80.988 (16)	Np1^ii^—Se1—Np1^vii^	101.074 (17)
Se2^vi^—Np1—Se1	160.988 (17)	Np1—Se1—Np1^vii^	98.541 (17)
Se2^vii^—Np1—Se1	74.905 (16)	Cu1^i^—Se2—Np1^vi^	77.53 (2)
Se2—Np1—Se1	86.315 (17)	Cu1^i^—Se2—Np1^v^	109.10 (3)
Se1^viii^—Np1—Se1	82.962 (10)	Np1^vi^—Se2—Np1^v^	100.770 (17)
Se2^ix^—Np1—Se1	121.133 (17)	Cu1^i^—Se2—Np1	146.34 (3)
Se2^vi^—Np1—Se1^v^	76.958 (16)	Np1^vi^—Se2—Np1	88.085 (16)
Se2^vii^—Np1—Se1^v^	141.588 (16)	Np1^v^—Se2—Np1	103.371 (19)
Se2—Np1—Se1^v^	72.469 (16)	Cu1^i^—Se2—Np1^xi^	79.48 (2)
Se1^viii^—Np1—Se1^v^	78.926 (17)	Np1^vi^—Se2—Np1^xi^	148.12 (2)
Se2^ix^—Np1—Se1^v^	144.944 (16)	Np1^v^—Se2—Np1^xi^	107.484 (18)
Se1—Np1—Se1^v^	84.479 (14)	Np1—Se2—Np1^xi^	99.254 (17)

Symmetry codes: (i) −*x*+1, −*y*+1, −*z*; (ii) −*x*, *y*+1/2, −*z*+1/2; (iii) −*x*, −*y*+1, −*z*; (iv) *x*, *y*+1, *z*; (v) *x*, −*y*+1/2, *z*−1/2; (vi) −*x*+1, −*y*, −*z*; (vii) *x*, −*y*+1/2, *z*+1/2; (viii) −*x*, *y*−1/2, −*z*+1/2; (ix) −*x*+1, *y*−1/2, −*z*+1/2; (x) *x*, *y*−1, *z*; (xi) −*x*+1, *y*+1/2, −*z*+1/2.
